# Editorial for Life Special Issue Book Cellular and Functional Response to Hypoxia

**DOI:** 10.3390/life13010005

**Published:** 2022-12-20

**Authors:** Jean-Paul Richalet

**Affiliations:** Laboratory of Cellular and Functional Responses to Hypoxia, University of Paris 13, 93017 Bobigny, France; richalet@univ-paris13.fr

Hypoxia is a current research topic in biology, physiology, and medicine. The effects of hypoxia and the adaptive and maladaptive responses to the hypoxic stress are extensively explored in the domains of altitude, exercise, pulmonary and cardiovascular medicine, inflammation, immunity, cancer, and metabolic diseases. However, the molecular machinery that regulates the activity of certain genes in response to hypoxia is still not explored. This Special Issue “Cellular and Functional Response to Hypoxia” presents 14 papers dealing with all aspects of responses to hypoxia, at the molecular, cellular, and integrative levels [1,2,3]. First, cellular signaling in hypoxia is addressed. Then, ventilatory, cardiovascular, and hematological responses are explored, with special attention on the effects of exercise in hypoxia. Finally, the effects of altitude on some cardiovascular and pulmonary diseases are presented. Altogether, this volume offers a large spectrum of current research on the effects of altitude hypoxia in the fields of health and disease. Thank you again to all contributors for this Special Issue of *Life*.
